# HIDEA syndrome is caused by biallelic, pathogenic, rare or founder *P4HTM* variants impacting the active site or the overall stability of the P4H‐TM protein

**DOI:** 10.1111/cge.14203

**Published:** 2022-08-19

**Authors:** Minna Kraatari‐Tiri, Leila Soikkonen, Matti Myllykoski, Yalda Jamshidi, Ehsan G. Karimiani, Jonna Komulainen‐Ebrahim, Hanna Kallankari, Cyril Mignot, Christel Depienne, Boris Keren, Marie‐Christine Nougues, Zahra Alsahlawi, Antonio Romito, Javier Martini, Mehran B. Toosi, Christopher J. Carroll, Kornelia Tripolszki, Peter Bauer, Johanna Uusimaa, Aida M. Bertoli‐Avella, Peppi Koivunen, Elisa Rahikkala

**Affiliations:** ^1^ PEDEGO Research Unit University of Oulu Oulu Finland; ^2^ Department of Clinical Genetics and Medical Research Center Oulu University Hospital Oulu Finland; ^3^ Department of Biomedicine University of Bergen Bergen Norway; ^4^ Genetics Section Molecular and Clinical Sciences Research Institute, St. George's, University of London London UK; ^5^ Department of Genetics Next Generation Polyclinic Mashhad Iran; ^6^ Department of Children and Adolescents and Medical Research Center Oulu University Hospital Oulu Finland; ^7^ APHP.Sorbonne Université, Département de Génétique, Hôpital Armand Trousseau and Groupe Hospitalier Pitié‐Salpêtrière Centre de Référence Déficiences Intellectuelles de Causes Rares Paris France; ^8^ Département de Génétique, Groupe Hospitalier Pitié‐Salpêtrière APHP.Sorbonne Université Paris France; ^9^ Département de Neuropédiatrie APHP.Sorbonne Université, Hôpital Trousseau Trousseau France; ^10^ Department of Pediatrics Salmaniya Medical Complex Kingdom of Bahrain Bahrain; ^11^ Department of Medical Reporting and Genomics Centogene GmbH Rostock Germany; ^12^ Department of Pediatrics School of medicine, Mashhad University of Medical Sciences Mashhad Iran; ^13^ Biocenter Oulu University of Oulu Oulu Finland; ^14^ Faculty of Biochemistry and Molecular Medicine Oulu Centre for Cell‐Matrix Research, University of Oulu Oulu Finland

**Keywords:** genes, HIDEA, intellectual disability, P4HTM, recessive

## Abstract

HIDEA syndrome is caused by biallelic pathogenic variants in *P4HTM*. The phenotype is characterized by muscular and central hypotonia, hypoventilation including obstructive and central sleep apneas, intellectual disability, dysautonomia, epilepsy, eye abnormalities, and an increased tendency to develop respiratory distress during pneumonia. Here, we report six new patients with HIDEA syndrome caused by five different biallelic *P4HTM* variants, including three novel variants. We describe two Finnish enriched pathogenic *P4HTM* variants and demonstrate that these variants are embedded within founder haplotypes. We review the clinical data from all previously published patients with HIDEA and characterize all reported *P4HTM* pathogenic variants associated with HIDEA in silico. All known pathogenic variants in *P4HTM* result in either premature stop codons, an intragenic deletion, or amino acid changes that impact the active site or the overall stability of P4H‐TM protein. In all cases, normal P4H‐TM enzyme function is expected to be lost or severely decreased. This report expands knowledge of the genotypic and phenotypic spectrum of the disease.

## Graphical abstract

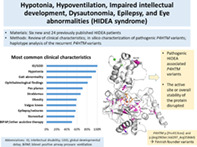

## 1 INTRODUCTION

Hypotonia, hypoventilation, impaired intellectual development, dysautonomia, epilepsy, and eye abnormalities (HIDEA) (OMIM #618493) is an autosomal recessive neurodevelopmental disorder caused by biallelic pathogenic variants in prolyl 4‐hydroxylase, transmembrane (*P4HTM*). Functional characterization of the pathogenic *P4HTM* variants revealed an improper folding of the corresponding protein, suggesting a loss‐of‐function disease mechanism.^1^, ^2^

Eukaryotic prolyl 4‐hydroxylases (P4Hs) are key enzymes in the synthesis of collagens and the regulation of oxygen homeostasis.^3^ P4H‐TM is localized to the endoplasmic reticulum (ER) membrane. It contains a short N‐terminal cytoplasmic region, a transmembrane helix, and within the ER lumen an EF‐hand domain and a P4H domain. The active site is composed of two His and one Asp residues that together with the co‐substrate 2‐oxoglutarate coordinate the Fe^2+^ which is central to the P4H‐TM activity. Recently, P4H‐TM was found to participate in gliotransmission in astrocytes, raising the question of whether this might be linked to the intellectual disability (ID) phenotype observed in HIDEA.^4^

To the authors' knowledge, 24 HIDEA patients with 12 different disease‐associated *P4HTM* variants have been described in the medical literature.^1^, ^2^, ^5^, ^6^, ^7^, ^8^ Here, we review the clinical and molecular data from all the published patients and describe six previously unpublished patients with HIDEA caused by five different biallelic pathogenic *P4HTM* variants.

## 2 MATERIALS AND METHODS

### 2.1 Patient recruitment

Patients were enrolled from three centers: Oulu University Hospital, Oulu, Finland (Families 1, 3–4), Next Generation Polyclinic, Mashhad, Iran (Families 2 and 6), and University of Sorbonne, Paris, France (Family 5). Families 1, 3, and 4 were identified after genetic testing or databank query in Centogene (Rostock, Germany). Families 2 and 6 were identified through GeneMatcher,^9^ and Family 5 was identified through ERN‐ITHACA call for collaboration. Detailed clinical data of Patients 1–6 and pedigrees are provided in the supplementary case histories and Figure S1.

### 2.2 Molecular genetics

Genomic DNA was extracted from peripheral blood samples using standard methods. WES was performed for all index cases and the parents of Families 1 and 3. Targeted Sanger sequencing was used for segregation analysis of the identified *P4HTM* variants in siblings of Family 1 and 2, and the parents of Families 2, 4, 5, and 6. Details of WES and haplotype analysis are included in the supplementary methods.

## 3 RESULTS

### 3.1 Clinical data

**FIGURE 1:**
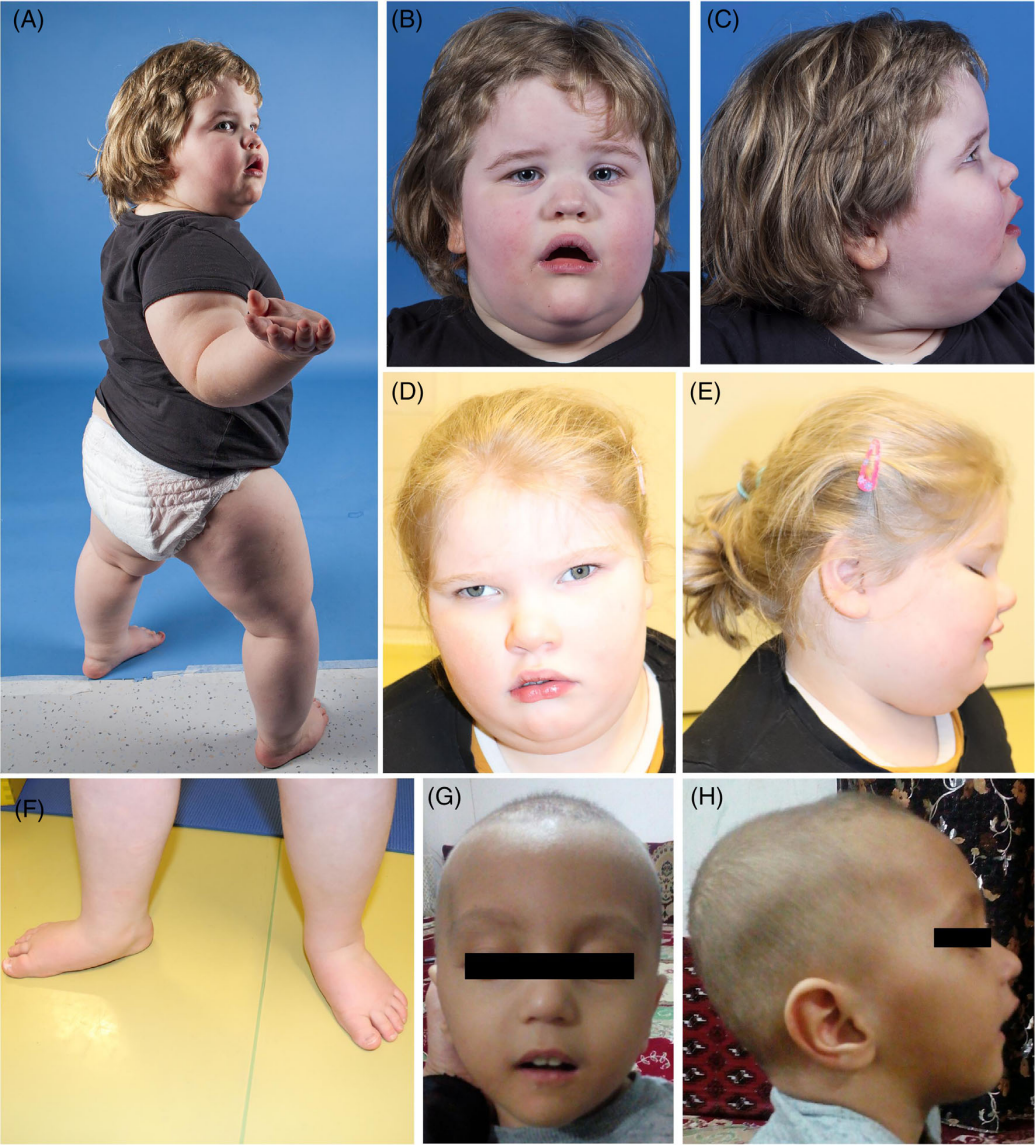
Clinical characteristics of the patients. All the patients show facial hypotonia with an open‐mouth appearance, tented upper lip vermilion, and a low nasal bridge. Strabismus (D), retrognathia (G, H), and pes planus (A, F) are shown [Colour figure can be viewed at wileyonlinelibrary.com]

We review the clinical details of both the new (*N* = 6) and previously reported HIDEA patients (*N* = 24; Figure 1, Table 1, Table S1).

**TABLE 1:** Main clinical features of Patients 1–6 described in this report, and the clinical features of previously published HIDEA patients

	Patient 1	Patient 2	Patient 3	Patient 4	Patient 5	Patient 6	Freq in this report	Freq in publ cases	Freq in all cases
Obesity	Yes	No	Yes	Yes	Yes	No	4/6	13/23	59% (17/29)
Hypotonia	Yes	Yes	Yes	No	Yes	Yes	5/6	24/24	97% (29/30)
ID/GDD	Yes	Yes	Yes	Yes	Yes	Yes	6/6	24/24	100% (30/30)
Learned to walk	Yes	No	Yes	Yes	Yes	No	4/6	11/19	60% (15/25)
Verbal	Yes	No	Yes	Yes	Yes	Yes	5/6	4/14	45% (9/20)
Epilepsy/seizures	No	Yes	Yes	Yes	Yes	Yes	5/6	12/24	57% (17/30)
Nystagmus	No	No	Yes	No	No	NA	1/6	7/20	31% (8/26)
Strabismus	No	No	Yes	No	Yes	Yes	3/6	13/18	67% (16/24)
Other ophthalmological findings	No	No	Yes	Yes	No	NA	2/5	20/22	81% (22/27)
MRI brain abnormalities	No	Yes	No	No	No	No	1/6	5/13	32% (6/19)
Obstructive sleep apnea	Yes	Yes	NA	No	NA	No	2/4	7/17	43% (9/21)
Central sleep apnea	Yes	Yes	NA	No	NA	No	2/4	8/18	45% (10/22)
BiPAP or other assistive therapy	Yes	NA	No	No	No	No	1/5	12/21	50% (13/26)
Parasomnia	No	No	NA	Yes	Yes	Yes	3/5	5/17	36% (8/22)
Impaired thermoregulation	Yes	Yes	No	No	No	No	2/6	4/16	27% (6/22)
Constipation	No	No	Yes	No	No	Yes	2/6	7/20	35% (9/26)
Valgus knees	No	No	Yes	No	No	No	1/6	6/6	58% (7/12)
Varus knees	Yes	No	No	No	No	Yes	2/6	0/6	17% (2/12)
Flexion/extension of the knees^a^	Yes	No	NA	No	No	NA	1/4	5/5	67% (6/9)
Pes planus	Yes	No	Yes	No	No	No	2/6	8/8	71% (10/14)
Gait abnormality	Yes	NA	Yes	Yes	No	NA	3/4	5/5	89% (8/9)

Abbreviations: freq, frequency; GDD, global developmental delay; ID, intellectual disability; NA, not available; publ, published.

^a^ When walking.

Twenty‐three patients are alive (age at last examination from 9 months to 59 years) and seven are deceased (age of death 7 months–61 years). The most common cause of death was respiratory tract infection (*N* = 4/7, 57%). Nineteen patients are male, and 11 are female. All patients have global developmental delay (DDD)/ID (*N* = 30/30, 100%). Common features include hypotonia (*N* = 29/30, 97%), epilepsy (*N* = 17/30, 57%), strabismus (*N* = 16/24, 67%), nystagmus (*N* = 8/26, 31%) or other ophthalmological abnormalities such as abnormal eye movements, cortical blindness, refractive errors, or achromic fundi (*N* = 22/27, 81%). Central (*N* = 10/22, 45%) and/or obstructive sleep apneas (*N* = 9/21, 43%) are common associated features and many patients (*N* = 13/26, 50%) require bilevel positive airway pressure ventilation (BiPAP) or other forms of respiratory support.

Most patients (*N* = 17/29, 59%) are or have been obese (>95th percentile). Six patients (*N* = 6/22, 27%) show dysautonomia of thermoregulation, including recurrent hypothermia or hyperthermia and reduced sweating. Facial dysmorphisms such as tented upper lip vermilion and low nasal bridge, are common (*N* = 22/23, 97%), but individual features vary. Brain MRIs are normal in most patients (*N* = 13/19, 68%), but three have brain atrophy, two have abnormalities of the white matter, and one patient has both. A majority of patients learned to walk (*N* = 15/25, 60%), walking age ranging from 18 months to 4 years. Patients who have achieved independent walking frequently present with gait abnormalities (*N* = 8/9, 89%). The age of first words ranges from 1 to 4 years, while 11 patients out of 20 (55%) were nonverbal at the time of study.

### 3.2 Molecular genetics

Five *P4HTM* (NM_177939.3) variants were observed in a homozygous or compound heterozygous state in the six patients in the current study: c.1238C>T, p.(Pro413Leu); c.1371G>A, p.(Trp457*); c.1073G>A, p.(Arg296Ser;Val297_Arg358del); c.1082C>T, p.(Thr361Ile); and c.934G>A, p.(Glu312Lys).

Haplotype analysis of the two recurrent variants revealed shared haplotypes extending approximately 7 Mb around the *P4HTM* p.(Pro413Leu) variant and 6.9 Mb around the *P4HTM* p.(Arg296Ser;Val297_Arg358del) variant.

**FIGURE 2:**
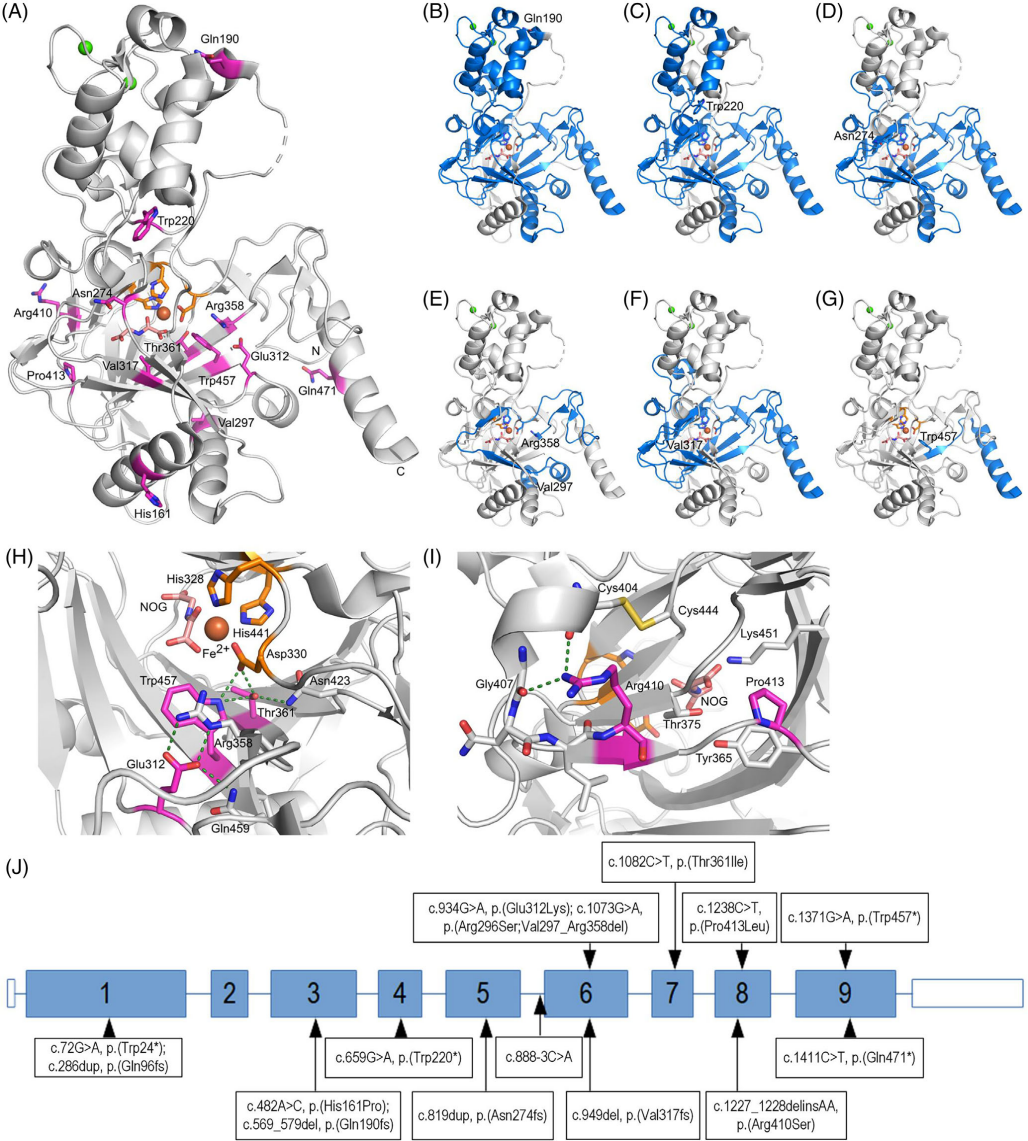
The residues targeted by the P4H‐TM HIDEA variants are presented in a cartoon and stick model of the P4H‐TM crystal structure. (A) An overview of the P4H‐TM crystal structure (residues 107–481). The side chains of the residues impacted by different variants are shown as sticks and colored magenta. The active site residues and Fe^2+^ are shown in orange and the 2‐oxoglutarate analog N‐oxalylglycine (NOG) in pink. Ca^2+^ ions are shown as green spheres. The N‐ and C‐termini and the variant residues are labeled. The deleted regions are shown in blue for the (B) Gln190Leufs*9, (C) Trp220*, (D) Asn274Glufs*11, (E) Arg296Ser;Val297_Arg358del, (F) Val317Phefs*30, and (G) Trp457* variants. The environment and interacting residues of (H) Glu312, Thr361, and Trp457 and (I) Arg410 and Pro413 are presented as a cartoon model where the interacting residues are shown as sticks and labeled. Hydrogen bonds and electrostatic interactions generated by the targeted residues are shown as green dashed lines. (G) Pathogenic *P4HTM* variants reported in the current study (above the gene) and in the literature (underneath the gene) are distributed throughout the gene (NM_177939.3). Similar HIDEA phenotype can be caused by pathogenic missense, nonsense, splice‐site, and frameshift variants, as well as in‐frame deletions [Colour figure can be viewed at wileyonlinelibrary.com]

We characterized the HIDEA causing variants using the recently published multiple sequence alignments and crystal structure showing the residues from 107 to 481 of the prevalent 502‐residue form of P4H‐TM (Figure 2A).^10^ Nonsense, frameshift, and in‐frame deletion variants in *P4HTM* (Figure 2B–G) are likely be degraded by nonsense‐mediated decay, or degraded due to protein misfolding and will not retain any P4H‐TM enzyme activity. The *P4HTM* missense variants p.(Thr361Ile) and p.(Pro413Leu) are residues conserved in P4Hs, while p.(Glu312Lys) is conserved in collagen prolyl 4‐hydroxylases but not in the hypoxia‐inducible factor prolyl 4‐hydroxylases. Glu312 and Thr361 are in the vicinity of the active site and interact with central active site residues (Figure 2H). The missense substitutions would lose these interactions and disrupt the positions of the central residues. The Pro413 side chain is positioned in a hydrophobic pocket near Lys451 and Tyr365 (Figure 2I). Leucine substitution here would disrupt the neighboring residues that help to coordinate the co‐substrate 2‐oxoglutarate (Figure 2I). Pathogenic missense variants resulting in the loss of the conserved residues are likely to decrease or completely abolish P4H‐TM enzyme activity.

Details of pathogenic *P4HTM* variants (Figure 2J), their in silico characterization, and haplotype analysis are provided in the supplementary results and Tables S2 and S3.

## 4 DISCUSSION

Here, we report six new unrelated patients and review the clinical details of all 24 previously published HIDEA patients. The phenotype of the patients identified in the current study is comparable to the phenotype described in the literature.^1^, ^2^, ^5^, ^6^, ^7^, ^8^ One patient in this study has dystonia, which has not previously been described in HIDEA. Dystonia is a movement disorder thought to result from an abnormality or damage to the basal ganglia or other brain regions controlling movement. The patient had generalized brain atrophy and cerebellar atrophy has previously been associated with dystonia.^11^ In addition, P4HTM is expressed in the basal ganglia,^12^ and P4THM deficiency may predispose to basal ganglia dysfunction leading to dystonia. Further research is needed to confirm the possible association between HIDEA and dystonia.

One third of all reported patients had history of pneumonias, and respiratory tract infection were the most common cause of death. Almost half of the HIDEA patients had central and/or obstructive sleep apneas and half required BiPAP treatment at nights or during respiratory infections. Thus, performing polysomnography and assessing the need for noninvasive ventilatory support is advisable.

The clinical presentation of HIDEA is variable, even for the same variant, and no clear genotype–phenotype correlation has been observed.^2^, ^5^ In the current study, patients with pathogenic homozygous *P4HTM* p.Glu312Lys missense and homozygous *P4HTM* p.Trp457* nonsense variants had similarly severe ID, confirming that both missense and truncating variants in *P4HTM* can result in a severe HIDEA phenotype. It is likely that background genetic factors, environmental factors, and stochastic factors modify the severity of the phenotype.

Haplotype analysis of the two recurring *P4HTM* variants, p.(Arg296Ser;Val297_Arg358del) and p.(Pro413Leu), revealed shared haplotypes, suggesting that both *P4HTM* variants are embedded within founder haplotypes. This is often seen in the Finnish population due to historical population bottlenecks, genetic drift events, and recent population expansion. In contrast, we identified two pathogenic novel and unique homozygous *P4HTM* variants, p.(Trp457*) and p.(Glu312Lys), in two consanguineous Iranian families, where consanguineous marriages are common increasing the risk for children with autosomal recessive disorders.

All known pathogenic variants in *P4HTM* are predicted to lose or decrease P4H‐TM enzyme activity. 5′ prime nonsense variants and the deletion of exon 6 lose critical parts of the P4H domain. Nonsense variants at positions 457 and 471 lose the ER retention signal and disrupt protein folding. Missense variants of conserved amino acids of the P4H domain disrupt the active site coordination or the binding of substrate or co‐substrate. Other missense variants are predicted to disrupt protein folding.

To the authors' knowledge, *P4HTM* p.(Glu312Lys), p.(Thr361Ile), and p.(Trp457*) variants have not previously been reported as disease‐causing, hence expanding the genotypic spectrum of the disease. To date, including the cases reported in the present study, there are 15 different pathogenic variants of *P4HTM* reported to cause HIDEA syndrome.^1^, ^2^, ^5^, ^6^, ^7^, ^8^

## 5 CONCLUSIONS

HIDEA syndrome is a recognizable neurodevelopmental disorder caused by pathogenic rare or founder *P4HTM* variants that are likely to disrupt the P4H‐TM activity. Greater knowledge of the genotypic and phenotypic spectrum of HIDEA will support the development of tailored therapies benefiting the patients.

## AUTHOR CONTRIBUTIONS

Conceptualization: Leila Soikkonen, Minna Kraatari‐Tiri, Elisa Rahikkala. Writing—original draft: Leila Soikkonen, Minna Kraatari‐Tiri, Matti Myllykoski, Elisa Rahikkala. Writing—review and editing: Minna Kraatari‐Tiri, Leila Soikkonen, Matti Myllykoski, Yalda Jamshidi, Ehsan G. Karimiani, Jonna Komulainen‐Ebrahim, Hanna Kallankari, Cyril Mignot, Boris Keren, Hanna Kallankari, Marie‐Christine Nougues, Zahra Alsahlawi, Antonio Romito, Javier Martini, Mehran B. Toosi, Christopher J. Carroll, Kornelia Tripolszki, Peter Bauer, Johanna Uusimaa, Aida M. Bertoli‐Avella, Peppi Koivunen, Elisa Rahikkala.

## CONFLICT OF INTEREST

Antonio Romito, Javier Martini, Kornelia Tripolszki, Peter Bauer, Aida M. Bertoli‐Avella are employees of CENTOGENE GmbH. Other authors declare no conflicts of interest.

### PEER REVIEW

The peer review history for this article is available at https://publons.com/publon/10.1111/cge.14203.

## ETHICS STATEMENT

The study is approved by the Ethics Committee of the Northern Ostrobothnia Hospital District (EETTMK: 186/2020). Written informed consent was obtained from all parents or guardians of the patients. Written informed consent was obtained to publish patient photos.

## Supporting information


**Appendix S1** Supporting Information

**Table S1** Clinical characteristics of patients described in this report (Patients 1–6) and all the previously published HIDEA patients to date.

**Table S3** Results of the haplotype analysis showing the shared haplotype around the *P4HTM* (NM_177939.3) p.(Pro413Leu) variant and *P4HTM* p.(Arg296Ser;Val297_Arg358del) variant. The shared haplotype extended approximately 7 Mb (GRCh38 g.3:45758441–52 818 394) around the *P4HTM* (NM_177939.3) p.(Pro413Leu) variant and approximately 6.9 Mb (GRCh38 g.3:46372893–53 235 071) around the *P4HTM* (NM_177939.3) p.(Arg296Ser;Val297_Arg358del) variant.

**Video S1** The gait of Patient 1. The video demonstrates the typical waddling gait associated with HIDEA syndrome.

**Video S2** Dystonic movements of Patient 2.

## Data Availability

The data that supports the findings of this study are available in the supplementary material of this article.
