# Supplementary material for: HIDEA syndrome is caused by biallelic, pathogenic, rare or founder P4HTM variants impacting the active site or the overall stability of the P4H‐TM protein
Source: Clin Genet. 2022 Aug 19;102(5):444–50. doi: 10.1111/cge.14203 (PMC9804808; doi:10.1111/cge.14203)
Supplement: Supplementary file 1 — Appendix S1 Supporting Information [file CGE-102-444-s004.docx]

**SUPPLEMENTARY MATERIAL**

**HIDEA syndrome is caused by biallelic, pathogenic, rare or founder *P4HTM* variants impacting the active site or the overall stability of the P4H-TM protein**

Minna Kraatari-Tiri^1,2,†^, Leila Soikkonen^1,2†^, Matti Myllykoski^3†^, Yalda Jamshidi^4†^, Ehsan G. Karimiani^4,5^, Jonna Komulainen-Ebrahim^1,6^, Hanna Kallankari^1,6^, Cyril Mignot^7^, Boris Keren^8^, Marie-Christine Nougues^9^, Zahra Alsahlawi^10^, Antonio Romito^11^, Javier Martini^11^, Mehran B. Toosi^12^, Christopher J. Carroll^4^, Kornelia Tripolszki^11^, Peter Bauer^11^, Johanna Uusimaa^1,6^, Aida M. Bertoli-Avella^11^, Peppi Koivunen^13,14^, Elisa Rahikkala^1,2^

^1^PEDEGO Research Unit, University of Oulu, Oulu, Finland

^2^Department of Clinical Genetics and Medical Research Center, Oulu University Hospital, Oulu, Finland

^3^Department of Biomedicine, University of Bergen, Bergen, Norway

^4^Genetics Section, Molecular and Clinical Sciences Research Institute, St George's, University of London, London, UK

^5^Next Generation Polyclinic, Mashhad, Iran

^6^Department of Children and Adolescents and Medical Research Center, Oulu University Hospital, Oulu, Finland

^7^APHP.Sorbonne Université, Département de Génétique, Hôpital Armand Trousseau and Groupe Hospitalier Pitié-Salpêtrière, Centre de Référence Déficiences Intellectuelles de Causes Rares, Paris, France

^8^APHP.Sorbonne Université, Département de Génétique, Groupe Hospitalier Pitié-Salpêtrière, Paris, France

^9^APHP.Sorbonne Université, Service de Neuropédiatrie, Hôpital Trousseau, Trousseau, France

^10^Salmaniya Medical Complex, Kingdom of Bahrain, Bahrain

^11^Centogene GmbH, 18055 Rostock, Germany

^12^Department of Pediatrics, School of medicine, Mashhad University of Medical Sciences, Mashhad, Iran.

^13^Biocenter Oulu, University of Oulu, Oulu, Finland

^14^Faculty of Biochemistry and Molecular Medicine, Oulu Centre for Cell-Matrix Research, University of Oulu, Oulu, Finland

^†^ These authors contributed equally

**Supplementary Case Histories of Patients 1–6**

**Patient 1** is a 4-year-old boy (Figure 1A–C; Family 1, Supplementary Figure 1). He is the fifth child of consanguineous Finnish parents. He was born at term with normal weight and height. The Apgar scores at 1/5/10 minutes were 9/10/10. During the pregnancy, his mother developed insulin-controlled gestational diabetes, high blood pressure and hepatogestosis and was induced at 37+5 weeks. Immediately following birth, he presented with central cyanosis, polycythemia, hypothermia (35.1⁰C) and bradycardia (70‒80 beats per minute) and was monitored at the neonatal intensive care unit. He recovered quickly and was discharged from the hospital at the age of three days. He has a global developmental delay (GDD), as well as a speech and motor delay. He learned to walk at the age of 19 months and saying individual words at approximately 2 years. He has a waddling gait (Supplementary video 1). He is morbidly obese and has an insatiable appetite. At the age of 3 years and 9 months his height was 109.23 cm (98^th^ percentile), weight 46.6 kg (>99.6^th^ percentile), body mass index (BMI) 39.1, BMI-standard deviation score (SDS) 5.6 and head circumference 55.8 cm (99.6^th^ percentile). He understands commands and expresses himself with vocalizations and around 30 words. He has macrosomic features and mild facial dysmorphisms. Magnetic resonance imaging (MRI) of the brain and abdomen is unremarkable. Polysomnography shows highly abnormal results that are diagnostic for both obstructive and central apnea, and bilevel positive airway pressure ventilation (BiPAP) therapy has been started.

In previous genetic testing, testing of the Prader-Willi syndrome (15q11-q13 region) and 41-gene monogenic obesity panel (Blueprint Genetics, Espoo, Finland) were negative. Array CGH shows two regions of homozygosity in chromosomes 3 (3p22.3p14.3, 22 Mb, chr3:32348341–54901360, hg19) and 15 (15q25.1q25.3, 8.01 Mb, chr15:78370743–86382188, hg19) resulting from parental consanguinity.

Trio exome sequencing (ES) of the patient (Centogene, Rostock, Germany) shows a homozygous missense variant in the *P4HTM* gene (NM_177938.3): c.1238C>T, p.(Pro413Leu), rs757914897. The variant has been previously published in two HIDEA patients ^1^ and is classified as likely pathogenic according to the ACMG guidelines (PM1, PM2, PP3, and PP4). The variant is present in the Genome Aggregation Database (gnomAD, 3-49043570-C-T | gnomAD v2.1.1 | gnomAD (broadinstitute.org)), including no homozygotes and with a minor allele frequency (MAF) of 0.00056 in the Finns. In silico analysis predicts the variant to be pathogenic (Mutation Taster, SIFT, PolyPhen-2, Revel) and its CADD score is 28. Subsequent segregation analysis has confirmed autosomal recessive inheritance. The parents and four siblings are healthy and unaffected heterozygous carriers of the variant.

**Patient 2** is a 3-year-old boy (Figure 1G–H; Family 2, Supplementary Figure 1). He is the third child of Iranian consanguineous parents. The pregnancy and delivery were unremarkable. His birth weight was 2950 g and height 47 cm and the Apgar scores were 9/10. He has hypotonia, severe GDD and is unable to walk or speak single words at the age of 3 years. He was diagnosed with epilepsy at the age of 1 year and 9 months. He also has obstructive and central sleep apneas but polysomnography has not been performed. He has dysautonomia including spikes of fever and anhidrosis. He also has glucose-6-phosphate dehydrogenase deficiency and hypoplasia of the common hepatic ducts. He was diagnosed with axial dystonia with tremor in the extremities at the age of 21 months (Supplementary video 2). Dystonia was treated unsuccessfully with trihexyphenidyl and levodopa.

Physical examination has revealed a short stature (height 81 cm (<0.4^th^ percentile)), while weight was 10 kg (<0.4^th^ percentile). He has small cheeks, micrognathia, tented upper lip and open mouth. He also has contracture of third fingers, genu recurvatum, and hypermobility of the ankles. MRI of the brain shows generalized brain atrophy. Bilateral sharp waves can be seen in the electroencephalography (EEG).

ES was done on a research basis using Agilent SureSelect V6 and has identified a novel homozygous nonsense variant in the *P4HTM* gene (NM_177939.3): c.1371G>A, p.(Trp457Ter) classified as pathogenic (PVS1, and PM2). It is predicted as disease causing (Mutation Taster) in silico, its CADD score is 42, and it is not present in the gnomAD. His parents are healthy heterozygous carriers of the variant. Two older siblings do not have the variant.

**Patient 3** is a 6-year-old girl (Figure 1D–E; Family 3, Supplementary Figure 1). She is the second child of non-consanguineous Finnish parents. She was born at term with a normal weight and height, and the Apgar scores were 9/9. Her mother had metformin treatment for gestational diabetes during the pregnancy. The neonatal period was uneventful. She was admitted to the hospital because of an epileptic seizure at 1.5 months of age. Focal epilepsy was diagnosed, and treatment with levetiracetam was started. Because of ongoing seizures, pyridoxine was first tried, and then, topiramate was added with a good response at the age of 3 months. Topiramate was successfully withdrawn at the age of 2 years, and since then, she has been free of seizures on levetiracetam monotherapy.

She presents with moderate intellectual disability (ID) and muscular hypotonia. She learned to walk independently and could say individual words at the age of 3 years. She has a broad-based gait. She is morbidly obese and has an insatiable appetite. She has occasional temper tantrums during which she screams or throws objects. At the age of 5 years and 2 months her height was 116 cm (94^th^ percentile), weight 38.20 kg (99^th^ percentile), BMI 28.4, and head circumference 52 cm (50^th^ percentile). She understands simple commands and but does not have functional speech. She has strabismus and nystagmus. She has a tented upper lip, open-mouth appearance, and facial hypotonia (Figure 1 D–E). She has pes planus in her feet (Figure 1F). MRI of the brain is normal. Polysomnography shows some obstructive and central sleep apneas and snoring.

In previous genetic testing, array CGH shows two regions of homozygosity in chromosomes 3 (3p21.31p14.3, 11 Mb, chr3:45775018–56771251, hg19) and 7 (7q34q35, 6,5 Mb, chr7:140233585–146767404, hg19).

Reanalysis of trio ES (Centogene, Rostock, Germany) has identified a homozygous missense variant in the *P4HTM* (NM_177939.3): c.1073G>A, p.(Arg296Ser;Val297_Arg358del) GRCh38 g.3:49005046G>A, rs182812551). The variant has been previously described to cause HIDEA syndrome ^2^ and causes an in-frame deletion of exon 6. It is classified as pathogenic (PVS1, PM2, PP1, and PP3) according to the ACMG guidelines. The variant is present in the gnomAD, including no homozygotes and with a MAF of 0.0007983 in the Finns. In silico analysis predicts the variant to be disease causing (Mutation Taster, SIFT, Provean, Revel) and most probably affecting splicing (Human Splicing Finder), and its CADD score is 33. The parents are healthy carriers of the variant.

**Patient 4** is a 3-year-old girl. She is the first child of non-consanguineous Finnish parents (Family 4, Supplementary Figure 1). Her mother has narcolepsy but did not use any medication during pregnancy. Delivery after 38+6 weeks of gestation was unremarkable. She had a normal birth weight of 2735 g. The Apgar scores were 9/9/9. Her motor development was delayed, and she started walking independently at 22 months. At 3 years, she had mildly wide-based gait and mild balance problems but no need for physiotherapy. She learned to say single words at the age of 1 years. At 3 years, she spoke in short sentences and had inarticulate speech, but did not need speech therapy yet. However, occupational therapy was started because of deficits in executive functions, and fine motor and perceptive skills.

At 2 years of age, she started to have symptoms that her parents thought to be night terrors. During child’s first visit to the pediatric neurology clinic at 3 years of age, the night-time symptoms turned out to be short (10 s), stereotypic seizures with a hypermotor component. EEG showed epileptiform spikes in the left sentroparietal region and focal discharges in the right frontal region. Focal epilepsy was diagnosed, and treatment with sodium valproate was started because of multifocal findings in the EEG. She did not respond well for the first medication. After sodium valproate was changed for the lacosamide, the control of her seizures was achieved.

Ophthalmologic evaluation is otherwise normal but reveals mild myopia. MRI of the brain is normal. Polysomnography is otherwise normal; but shows a few mild obstructive respiratory events. She is mildly obese and has an excessive appetite. At the age of 3 years 4 months, her height was 101 cm (85^th^ percentile), weight 21.3 kg (99^th^ percentile), and head circumference 51.2cm (75^th^ percentile).

ES (Centogene, Rostock, Germany) has identified compound heterozygous variants in the *P4HTM* gene (NM_177938.3): c.1073G>A, p.(Arg296Ser;Val297_Arg358del), rs182812551 and c.1238C>T, p.(Pro413Leu), rs757914897. Sanger sequencing shows that the mother is a healthy heterozygous carrier of the *P4HTM* c.1238C>T, p.(Pro413Leu) variant and that the father is a healthy carrier of *P4HTM* c.1073G>A, p.(Arg358Gln) variant, confirming that the variants are in trans.

**Patient 5** is a 6-year-old boy and the first child of French consanguineous parents (Family 5, Supplementary Figure 1). He was born after a normal pregnancy at 41 gestational weeks. His birth weight was 3020 g, height 51 cm, and OFC 34 cm, and the Apgar scores were 10/10. He had a GDD. He started to walk at the age of 26 months and said his first words at the age of 2 years.

He developed atypical asymmetrical spasms at 2–3 months. He was diagnosed with focal epilepsy which was responsive to vigabatrine medication. EEG showed a persistently slow background. At the age of 6 years and 6 months, his height was 126 cm (91^st^ percentile), weight 36.2 kg (99^th^ percentile), and OFC 52 cm (20^th^ percentile). He has strabismus. He has a tented upper lip and enophtalmia. He has frequent awakenings at nights. Polysomnography has not been performed.

ES (Paris, France) has identified a homozygous variant in the *P4HTM* gene (NM_177939.3): c.1082C>T, p.(Thr361Ile). It is classified as pathogenic (PM1, PM2, and PP3). The variant is not present in the gnomAD. In silico analysis predicts the variant to be disease causing (Mutation Taster, SIFT, Polyphen-2, Provean, Revel) and its CADD score is 27. The parents are asymptomatic heterozygous carriers of this *P4HTM* c.1082C>T variant.

**Patient 6** is a 3-year-old girl. She is the fifth child of consanguineous Iranian parents (Family 6, Supplementary Figure 1). She was born after a normal pregnancy at 39 gestational weeks. Her birth weight was 2700 g, height 49 cm, and OFC 34 cm, and the Apgar scores were 9/10.

She presented at 2 years of age with no speech and inability to walk independently. At the age of 2.5 years, she had epileptic seizures which were controlled with phenobarbital medication. EEG showed non-specific scattered sharp waves. Brain MRI showed normal brain structures. She has frequent awakenings at nights.

In the clinical examination at the age of 3.5 years, her height is 86 cm (<0.4^th^ percentile), weight is 12 kg (2^nd^ percentile), and OFC is 47 cm (<0.4^th^ percentile). She presents with muscular hypotonia and severe intellectual disability. She is not able to walk, and she can only say two words. She has strabismus. She has varus in her knees and hypermobile ankle joints. She has mild facial dysmorphism, including a tented upper lip vermilion and a low nasal bridge.

The second child in her family had cerebral palsy and epilepsy and she died at the age of 2 years. Other older siblings have normal growth and development.

ES was done on a research basis using Agilent SureSelect V6 and has identified a homozygous variant in the *P4HTM* gene (NM_177939.3): c.934G>A, p.(Glu312Lys). It is classified as pathogenic (PM1, PM2, and PP3) according to the ACMG/AMP criteria. The variant is not present in the gnomAD. It is predicted as disease causing (Mutation Taster, SIFT, PolyPhen-2, Provean, Revel), and its CADD score is 31. Her parents are healthy heterozygous carriers of this variant.

Detailed clinical information of the patients described in this report and all the previously published HIDEA patients to date are collected in the Supplementary Table 1.


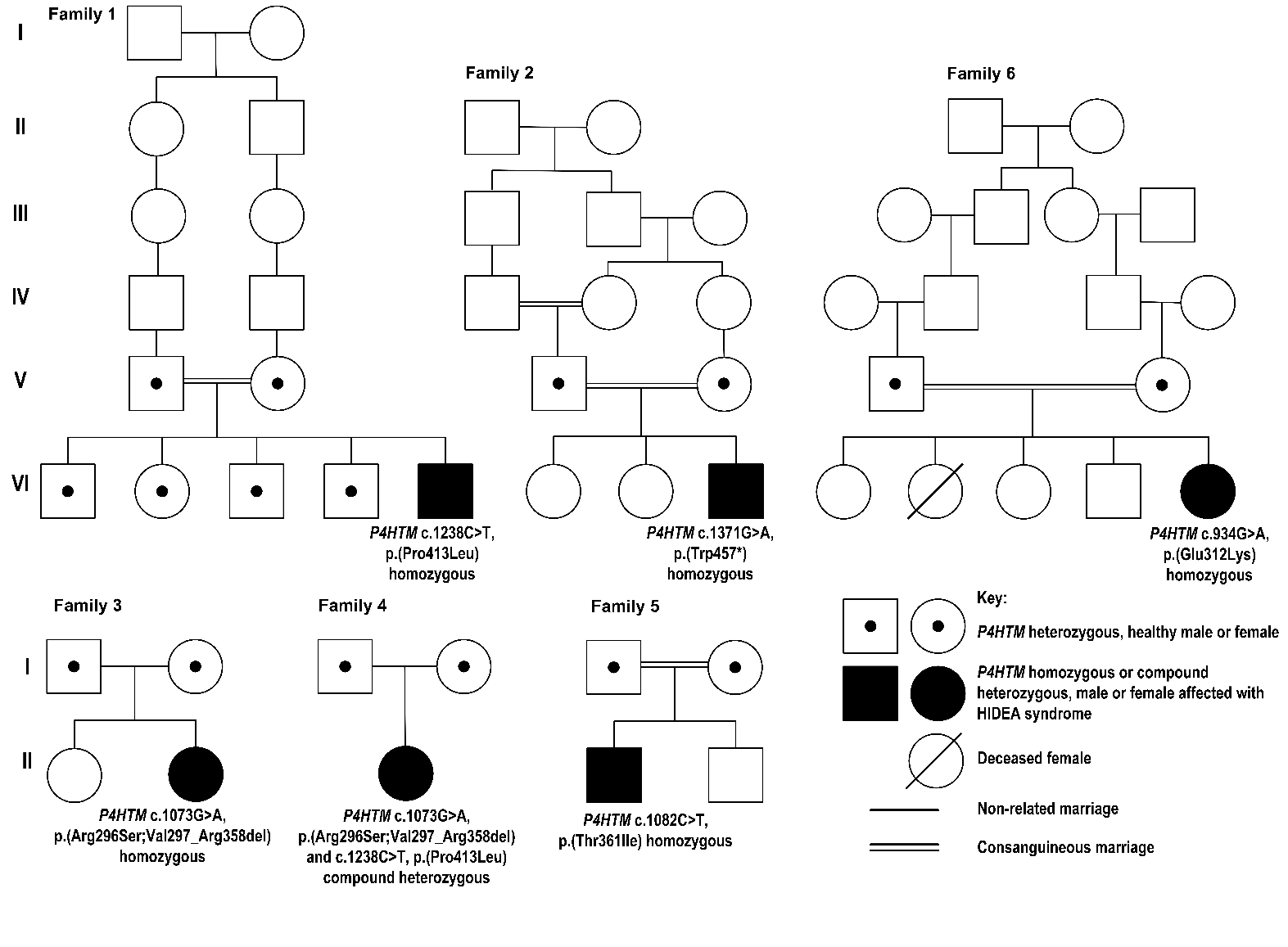
**Supplementary Figure 1.** Pedigrees of the families showing individuals either in two, five, or six generations (I–VI) and indicating the genotypes of the individuals who were available for genetic testing.

**Supplementary methods**

**Exome sequencing (ES)**

Genomic DNA was extracted from EDTA-treated blood samples using an automated QIAsymphony device and a Qiagen QIAsymphony DSP DNA Midi kit (Qiagen, Hilden, Germany).

ES was performed for all the index patients, and the parents of Families 1 and 3. Targeted Sanger sequencing was used for cosegregation analysis of the variant in four siblings of Family 1, and the parents of Families 4 and 5.

ES was performed as previously described ^3^. Briefly, the Nextera Rapid Capture Exome Kit (Illumina, San Diego, CA), the SureSelect Human All Exon kit (Agilent, Santa Clara, CA), the Twist Human Core Exome, or the Roche MedExome Kit (Roche, Basel, Switzerland) was used for enrichment, and a Nextseq500, HiSeq4000, or Novoseq 6000 (Illumina) platform was used to perform paired-end sequencing, with the average coverage targeted to at least 75x–100x or at least 98% of the target DNA covered by 20x. Variants of low quality were confirmed by Sanger sequencing according to the established criteria ^4^.

Centogene bio/databank was queried for rare homozygous or compound heterozygous variants in the *P4HTM* gene. At the time of the analysis, our data repository (CentoMD®) ^5^ contained ES/GS data from 39,210 unique families with 39,756 affected individuals. Rare variants with gnomAD frequencies ≤ 0.001, predicted to lead to loss of function (pLoF), or predicted as deleterious by in silico tools (phred Combined Annotation Dependent Depletion (CADD) score above 20) ^6^ were assessed. Only variants with satisfactory quality scores were considered (read depth ≥ 20, frequency ≥ 20 and quality score ≥ 220) ^4^.

The sequence variant nomenclature followed the guidelines of the standard Human Genome Variation Society (HGVS) ^7^. Variants were classified according to the guidelines of the American College of Medical Genetics (ACMG) as pathogenic (P), likely pathogenic (LP), or variant of unknown significance (VUS) ^8^.

***Haplotype analysis***

Haplotype analysis was performed using the WES data from three unrelated Finnish Patients 1, 3, and 4 who carried biallelic pathogenic *P4HTM* (NM_177939.3) p.(Arg296Ser;Val297_Arg358del) and p.(Pro413Leu) variants either in a homozygous or compound heterozygous state. Single nucleotide variants with a minor allele frequency (MAF) of maximum 0.1 were selected and analyzed in a region approximately 10 Mb around the *P4HTM* gene (GRCh38 g.3:43,812,829-54,198,790).

**Supplementary results**

***P4HTM variants identified in this study***

*P4HTM* c.1238C>T, p.(Pro413Leu) variant (GRCh38 g. 3:49006137C>T, rs757914897) identified in two Finnish families (Family 1 and 4) is present in the Genome Aggregation Database (gnomAD v.2.1.1, accessed on 27^th^ January 2022) with a MAF of 0.000555 in the Finnish population, including no homozygotes. The variant has previously been reported in two Finnish patients with severe-profound ID ^1^. According to the ACMG/AMP criteria ^8^, *P4HTM* c.1238C>T is classified as likely pathogenic (PM1, PM2, PP3, and PP4).

*P4HTM* c.1371G>A, p.(Trp457*) (GRCh38 g.3:49006769G>A) identified in an Iranian family (Family 2) is not present in gnomAD and has not been previously published. It is classified as pathogenic (PVS1, and PM2) according to the ACMG/AMP criteria.

*P4HTM* c.1073G>A, p.(Arg296Ser;Val297_Arg358del) (GRCh38 g.3:49005046G>A, rs182812551) identified in two Finnish families (Family 3 and 4) is present in gnomAD with a MAF of 0.00009198. The allele frequency is 21 times higher in Finns (0.0007983) than in non-Finnish Europeans (0.00003873), and there are no homozygous individuals in gnomAD. It is classified as pathogenic (PVS1, PM2, PP1, and PP3) according to the ACMG/AMP criteria.

*P4HTM* c.1082C>T, p.(Thr361Ile) variant (GRCh38 g.3:49005785C>T) identified in a French family (Family 5) is not present in gnomAD. It is classified as pathogenic (PM1, PM2, and PP3) according to the ACMG/AMP criteria.

*P4HTM* c.934G>A, p.(Glu312Lys) variant (GRCh38 g.3:49004907G>A) identified in an Iranian family (Family 6) is not present in gnomAD. It is classified as pathogenic (PM1, PM2, and PP3) according to the ACMG/AMP criteria.

Details of the identified variants are provided in the Supplementary Table 2.

| **Pathogenic variants in the P4HTM gene** | **Patient 1** | **Patient 2** | **Patient 3** | **Patient 4** | **Patient 5** | **Patient 6** |
| --- | --- | --- | --- | --- | --- | --- |
| Genomic position (GRCh38) | g.3:49006137C>T | g.3:49006769G>A | g.3:49005046G>A | g.3:49006137C>T, g.3:49005046G>A | g.3:49005785C>T | g.3:49004907G>A |
| Transcript | NM_177939.3 | NM_177939.3 | NM_177939.3 | NM_177939.3 | NM_177939.3 | NM_177939.3 |
| cDNA | c.1238C>T | c.1371G>A | c.1073G>A | c.1238C>T, c.1073G>A | c.1082C>T | c.934G>A |
| Genotype | Homozygous | Homozygous | Homozygous | Compound heterozygous | Homozygous | Homozygous |
| Exon | 8 | 9 | 6 | 8 and 6 | 7 | 6 |
| Protein change | p.(Pro413Leu) | p.(Trp457*) | p.(Arg296Ser;Val297_Arg358del) | p.(Pro413Leu), p.(Arg296Ser;Val297_Arg358del) | p.(Thr361Ile) | p.(Glu312Lys) |
| ACMG criteria | Likely pathogenic (PM1, PM2, PP3, PP4) | Pathogenic (PVS1, and PM2) | Pathogenic (PVS1, PM2, PP1, PP3) | Likely pathogenic (PM1, PM2, PP3, PP4), Pathogenic (PVS1, PM2, PP1, PP3) | Pathogenic (PM1, PM2, PP3) | Pathogenic (PM1, PM2, PP3) |
| Coding effect | Missense | Nonsense | In-frame deletion | Missense, in-frame deletion | Missense | Missense |
| Minor allele frequency in gnomAD | 4.8 * 10^-5^ | 0 | 9.2 * 10^-5^ | 4.8 * 10^-5^ , 9.2 * 10^-5^ | 0 | 0 |
| In silico predictions^6,9–13^ | Mutation Taster: disease causing, SIFT: deleterious, PolyPhen-2: probably damaging, Provean: Damaging, Revel: pathogenic, CADD score 28 | Mutation Taster: disease causing, CADD score 42 | Mutation Taster: disease causing, SIFT: deleterious, Provean: Damaging, Revel: pathogenic, Human Splicing Finder: most probably affecting splicing, CADD score 33 | Mutation Taster: disease causing, SIFT: deleterious, Provean: Damaging, Revel: pathogenic, CADD scores 28 and 33 | Mutation Taster: disease causing, SIFT: deleterious, PolyPhen-2: probably damaging, Provean: damaging, Revel: pathogenic, CADD score 27 | Mutation Taster: disease causing, SIFT: deleterious, PolyPhen-2: probably damaging, Provean: damaging, Revel: pathogenic, CADD score 31 |

**Supplementary Table 2.** Summary of the genetic variants identified in Patients 1-6.

***Haplotype analysis of two recurrent P4HTM variants***

We compared Patient 1 with a homozygous *P4HTM* (NM_177939.3) p.(Pro413Leu) variant to unrelated Patient 4 with compound heterozygous *P4HTM* (NM_177939.3) p.(Pro413Leu) and p.(Arg296Ser;Val297_Arg358del) variants. This analysis revealed a shared haplotype which extended approximately 7 Mb (GRCh38 g.3:45758441-52818394) around the *P4HTM* (NM_177939.3) p.(Pro413Leu) variant (Supplementary Table 3).

Similarly, Patient 3 with a homozygous *P4HTM* (NM_177939.3) p.(Arg296Ser;Val297_Arg358del) variant was compared with unrelated Patient 4 with compound heterozygous *P4HTM* (NM_177939.3) p.(Pro413Leu) and p.(Arg296Ser;Val297_Arg358del) variants. This analysis revealed also a shared haplotype which was approximately 6.9 Mb (GRCh38 g.3:46372893-53235071) around the *P4HTM* (NM_177939.3) p.(Arg296Ser;Val297_Arg358del) variant (Supplementary Table 3).

***In silico P4HTM* *variant characterization***

We characterized the HIDEA causing variants using the recently published multiple sequence alignments and crystal structure showing the residues from 107 to 481 of the prevalent 502-residue form of P4H-TM (Figure 2A) ^14^. The currently identified pathogenic *P4HTM* variants can be divided into four groups based on their predicted effect on the protein product: 1) large intragenic deletions, 2) truncations near the C-terminus, 3) single residue substitutions of residues conserved in P4Hs, or 4) single residue substitutions that disrupt the structural integrity of the protein.

The large deletions of the first group result in the predicted loss of the whole of the folded EF-hand and P4H domains p.(Gln96Profs*29) ^2^, the loss of most of these p.(Gln190Leufs*9) (Figure 2B) and p.(Trp220*) (Figure 2C) ^10^, or the loss of significant fractions of the P4H domain p.(Asn274Glufs*11) (Figure 2D), p.(Arg296Ser;Val297_Arg358del) (Figure 2E), and p.(Val317Phefs*30) (Figure 2F) ^2,15^. These protein variants are likely degraded by nonsense-mediated decay, or degraded due to protein misfolding, and will not retain any P4H-TM enzyme activity.

Group 2 protein truncation variants at the C-terminus remove the ER retention signal and most likely result in the mislocalization of the enzyme within the cell. The p.(Gln471*) variant at the C-terminal α-helix was also found to disrupt the folding of P4H-TM ^3^. The variant with a premature stop codon at codon 457 reported here is expected to share these properties (Figure 2G). In addition, Trp457 is one of the residues conserved among all P4Hs; it interacts with the conserved Arg358 and forms a hydrogen bond to Asp330 of the iron coordinating catalytic triad and to Thr361 (Figure 2H). The loss of Trp457 would probably decrease or eliminate P4H-TM activity by itself and in combination with the predicted C-terminal deletion it has the properties of both groups 2 and 3.

Of the other variant codons reported in this study, p.(Thr361Ile) and p.(Pro413Leu) are also on residues conserved in P4Hs, while Glu312Lys is conserved in C-P4Hs but not in HIF-P4Hs. Glu312 is found in the vicinity of the active site (Figure 2H) where it interacts with Arg358 and Gln459. A lysine substitution in this position would lose these interactions and disrupt the position of the central Arg358. The residues corresponding to both Glu312 and Arg358 were found to interact with the substrate peptide in the crystal structure of an algal P4H homologous to P4H-TM ^16^, suggesting that substrate binding would be disrupted in Glu312Lys variant of P4H-TM. Thr361 is located in the βIII strand of P4H-TM core and forms a hydrogen bond with Asp330, Asn423, and Trp457 (Figure 2H). An isoleucine substitution cannot form these interactions and will disrupt the coordination of Asp330. The Pro413 side chain is positioned in a hydrophobic pocket near Lys451 and Tyr365 (Figure 2I). Leucine substitution here would require a larger pocket and likely cause the displacement of the neighboring residues. Lys451 is a conserved basic residue that coordinates the cosubstrate 2-oxoglutarate, and Tyr365 also interacts with the cosubstrate via Thr375 (Figure 2I). Displacement of these residues is likely to lead to a reduced capacity for 2-oxoglutarate binding. Overall, the loss of the conserved residues in group 3 variants is likely to severely decrease or completely abolish P4H-TM enzyme activity.

The p.(His161Pro) variant was previously discovered as being able to introduce a disruptive proline in the middle of an α-helix and to severely disrupt the overall P4H-TM folding ^2,14^. The Arg410Ser variant was recently reported to be linked to HIDEA ^15^. Arg410 is found in the short β-strand βV and adopts a specific conformation at the surface of the protein, where one nitrogen of the guanidium group forms a hydrogen bond to the backbone carbonyl groups of Cys404 and Gly407 (Figure 2I). These hydrogen bonds stabilize the local secondary structure and possibly contribute to the formation of the disulfide bond between Cys404 and Cys444. The interactions are lost in the Arg410Ser variant. Arg410 is mostly conserved in vertebrate P4H-TMs but not in other P4Hs. These two missense variants are categorized into the fourth group. They do not have direct contact with the active site but instead seem to disrupt the structure and folding of P4H-TM. Some enzyme activity could be preserved here, depending on how severe the structural disruption is.

**References**

1. Järvelä I, Määttä T, Acharya A, et al. Exome sequencing reveals predominantly de novo variants in disorders with intellectual disability (ID) in the founder population of Finland. *Hum Genet*. 2021;140(7):1011-1029. doi:10.1007/s00439-021-02268-1

2. Rahikkala E, Myllykoski M, Hinttala R, et al. Biallelic loss-of-function P4HTM gene variants cause hypotonia, hypoventilation, intellectual disability, dysautonomia, epilepsy, and eye abnormalities (HIDEA syndrome). *Genet Med*. 2019;21(10):2355-2363. doi:10.1038/s41436-019-0503-4

3. Trujillano D, Bertoli-Avella AM, Kumar Kandaswamy K, et al. Clinical exome sequencing: results from 2819 samples reflecting 1000 families. *Eur J Hum Genet*. 2017;25(2):176-182. doi:10.1038/ejhg.2016.146

4. Bauer P, Kandaswamy KK, Weiss MER, et al. Development of an evidence-based algorithm that optimizes sensitivity and specificity in ES-based diagnostics of a clinically heterogeneous patient population. *Genet Med*. 2019;21(1):53-61. doi:10.1038/s41436-018-0016-6

5. Trujillano D, Oprea GE, Schmitz Y, Bertoli-Avella AM, Abou Jamra R, Rolfs A. A comprehensive global genotype-phenotype database for rare diseases. *Mol Genet Genomic Med*. 2017;5(1):66-75. doi:10.1002/mgg3.262

6. Rentzsch P, Witten D, Cooper GM, Shendure J, Kircher M. CADD: predicting the deleteriousness of variants throughout the human genome. *Nucleic Acids Res*. 2019;47(D1):D886-D894. doi:10.1093/nar/gky1016

7. den Dunnen JT, Dalgleish R, Maglott DR, et al. HGVS Recommendations for the Description of Sequence Variants: 2016 Update. *Hum Mutat*. 2016;37(6):564-569. doi:10.1002/humu.22981

8. Richards S, Aziz N, Bale S, et al. Standards and guidelines for the interpretation of sequence variants: a joint consensus recommendation of the American College of Medical Genetics and Genomics and the Association for Molecular Pathology. *Genet Med*. 2015;17(5):405-424. doi:10.1038/gim.2015.30

9. Schwarz JM, Cooper DN, Schuelke M, Seelow D. MutationTaster2: mutation prediction for the deep-sequencing age. *Nat Methods*. 2014;11(4):361-362. doi:10.1038/nmeth.2890

10. Ng PC, Henikoff S. Predicting deleterious amino acid substitutions. *Genome Res*. 2001;11(5):863-874. doi:10.1101/gr.176601

11. Adzhubei IA, Schmidt S, Peshkin L, et al. A method and server for predicting damaging missense mutations. *Nat Methods*. 2010;7(4):248-249. doi:10.1038/nmeth0410-248

12. Choi Y, Sims GE, Murphy S, Miller JR, Chan AP. Predicting the functional effect of amino acid substitutions and indels. *PLoS One*. 2012;7(10):e46688. doi:10.1371/journal.pone.0046688

13. Ioannidis NM, Rothstein JH, Pejaver V, et al. REVEL: An Ensemble Method for Predicting the Pathogenicity of Rare Missense Variants. *Am J Hum Genet*. 2016;99(4):877-885. doi:10.1016/j.ajhg.2016.08.016

14. Myllykoski M, Sutinen A, Koski MK, et al. Structure of transmembrane prolyl 4-hydroxylase reveals unique organization of EF and dioxygenase domains. *J Biol Chem*. 2021;296:100197. doi:10.1074/jbc.RA120.016542

15. Maddirevula S, Ben-Omran T, AlMureikhi M, et al. Further delineation of HIDEA syndrome. *Am J Med Genet A*. 2020;182(12):2999-3006. doi:10.1002/ajmg.a.61885

16. Koski MK, Hieta R, Hirsilä M, Rönkä A, Myllyharju J, Wierenga RK. The crystal structure of an algal prolyl 4-hydroxylase complexed with a proline-rich peptide reveals a novel buried tripeptide binding motif. *J Biol Chem*. 2009;284(37):25290-25301. doi:10.1074/jbc.M109.014050

**TABLE LEGENDS**

Supplementary Table 1. Clinical characteristics of patients described in this report (Patients 1–6) and all the previously published HIDEA patients to date.

Supplementary Table 3. Results of the haplotype analysis showing the shared haplotype around the *P4HTM* (NM_177939.3) p.(Pro413Leu) variant and *P4HTM* p.(Arg296Ser;Val297_Arg358del) variant. The shared haplotype extended approximately 7 Mb (GRCh38 g.3:45758441-52818394) around the *P4HTM* (NM_177939.3) p.(Pro413Leu) variant and approximately 6.9 Mb (GRCh38 g.3:46372893-53235071) around the *P4HTM* (NM_177939.3) p.(Arg296Ser;Val297_Arg358del) variant.

Supplementary video 1. The gait of Patient 1. The video demonstrates the typical waddling gait associated with HIDEA syndrome.

Supplementary video 2. Dystonic movements of Patient 2.
